# Convergent Synthesis of the Potent P2Y Receptor Antagonist MG 50-3-1 Based on a Regioselective Ullmann Coupling Reaction

**DOI:** 10.3390/molecules17032599

**Published:** 2012-03-05

**Authors:** Younis Baqi, Christa E. Müller

**Affiliations:** PharmaCenter Bonn, Pharmaceutical Institute, Pharmaceutical Chemistry I, Pharmaceutical Sciences Bonn (PSB), University of Bonn, An der Immenburg 4, D-53121 Bonn, Germany; Email: younis.baqi@uni-bonn.de

**Keywords:** anthraquinone, convergent synthesis, P2Y receptor antagonist, regioselective coupling, Ullmann reaction

## Abstract

MG 50-3-1 (**3**, trisodium 1-amino-4-{4-[4-chloro-6-(2-sulfophenylamino)-1,3,5-triazin-2-ylamino]-2-sulfophenylamino}-9,10-dioxo-9,10-dihydroanthracene 2-sulfonate) is the most potent and selective antagonist (IC_50_ 4.6 nM) for “P2Y_1_-like” nucleotide-activated membrane receptors in guinea-pig taenia coli responsible for smooth muscle relaxation. Full characterization of the compound, however, e.g., at the human P2Y_1_ receptor, which is a novel potential target for antithrombotic drugs, as well as other P2 receptor subtypes, has been hampered due to difficulties in synthesizing the compound in sufficient quantity. MG 50-3-1 would be highly useful as a biological tool for detailed investigation of signal transduction in the gut. We have now developed a convenient, fast, mild, and efficient convergent synthesis of **3** based on retrosynthetic analysis. A new, regioselective Ullmann coupling reaction under microwave irradiation was successfully developed to obtain 1-amino-4-(4-amino-2-sulfophenylamino)-9,10-dioxo-9,10-dihydro­anthracene 2-sulfonate (**8**). Four different copper catalysts (Cu, CuCl, CuCl_2_, and CuSO_4_) were investigated at different pH values of sodium phosphate buffer, and in water in the absence or presence of base. Results showed that CuSO_4_ in water in the presence of triethylamine provided the best conditions for the regioselective Ullmann coupling reaction yielding the key intermediate compound **8**. A new synthon (sodium 2-(4,6-dichloro-1,3,5-triazin-2-ylamino)benzenesulfonate, **13**) which can easily be obtained on a gram scale was prepared, and **13** was successfully coupled with **8** yielding the target compound **3**.

## 1. Introduction

Extracellular nucleotides such as ATP, ADP, UTP or UDP are the physiological ligands for P2 purinergic receptors, which are localized in the cell membrane [1,2,3,4]. They are subdivided into G protein-coupled or metabotropic P2Y receptors (GPCRs), and ligand-gated ion channels (LGICs) or ionotropic receptors, termed P2X. Both families, GPCRs as well as LGICs constitute important novel drug targets [1,2,3,4]. In recent years, it was estimated that up to 50% of available drugs act via stimulation or blocking of GPCRs [5]. The P2Y_1_ and the P2Y_12_ receptor, for example, both of which are activated by ADP, are involved in platelet activation and aggregation [6,7,8,9]. The P2Y_12_ receptor is already an established target of antithrombotic drugs like clopidogrel (Plavix^®^) and ticagrelor (Brilinta^®^) [10,11,12]. P2Y_1_ antagonists may also have potential as novel antithrombotic drugs [6,7,13,14]. “P2Y_1_-like” receptors have been described in a number of early publications in the field to mediate smooth muscle relaxation in the intestine. Later on it was found that the intestinal preparations expressed both, P2Y_1_ and P2Y_11_ receptors [15].

**Figure 1:**
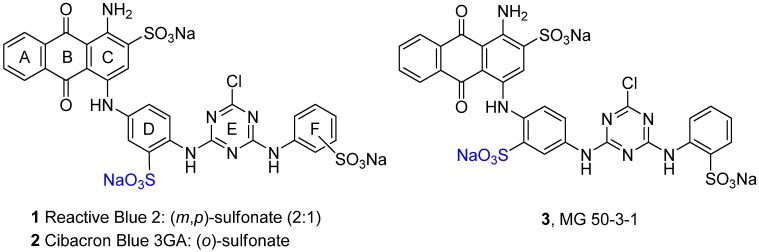
Structures of Reactive Blue 2 (RB-2, **1**), Cibacron Blue 3GA (**2**) and MG 50‑3‑1 (**3**).

The anthraquinone derivatives Reactive Blue 2 (RB-2, **1**, Figure 1) and Cibacron Blue 3GA (**2**, Figure 1) have been identified as new lead compounds in drug discovery [16,17,18,19]. They have been found to interact with a variety of nucleotide-binding proteins in the human body [17,19], including a number of different P2Y and P2X receptor subtypes as well as ecto-nucleotidases [17,19,20]. 

MG 50-3-1 (**3**, Figure 1) is an isomer of compound **2**, in which the sulfonate group in ring D is shifted from the *meta*- to the *ortho*-position with regard to the aminoanthraquinone substituent. Compound **3** has been described to be the most potent and selective antagonist for “P2Y_1_-like” receptors expressed in guinea-pig taenia coli, exhibiting an IC_50_ value of 4.6 nM [21]. Full characterization of the compound, however, has been hampered due to difficulties in synthesizing the compound in sufficient amounts, therefore, **3** could not be investigated so far at human P2Y_1_ receptors and at the other P2Y receptor subtypes. The compound could be a very useful pharmacological tool for basic research and target validation. The described linear synthesis, however, was reported to provide only 5.5% overall yield [21]. In our hands, the published approach was not successful at all. Therefore we developed a novel strategy for the preparation of the target compound **3**. Herein we describe a convergent access towards compound **3** which will eventually allow full characterization and *in vitro* as well as *in vivo* evaluation of the drug, and thus may provide information valuable for the development of antagonists for P2Y_1_ and P2Y_1_-like receptors. Furthermore, the compound will be a useful biological tool for investigating purinergic signalling, for example in the intestine.

## 2. Results and Discussion

Previous studies showed that the substitution pattern in the 4–position of the anthraquinone moiety plays a crucial role for the ability of the compounds to antagonize P2Y receptor subtypes, such as P2X1 and P2Y_1_-like [21], P2X2 [22], P2Y_2_ [23], and P2Y_12_ receptors [24,25] and to inhibit nucleoside triphosphate diphosphohydrolase (NTPDase) isoenzymes [26] and ecto-5’-nucleotidase [27]. Recently we developed a microwave-assisted Ullmann coupling reaction of bromaminic acid with a diverse range of aniline derivatives in the presence of elemental copper (Cu^0^) in sodium phosphate buffer [28,29]. In the present study we examined the impact of the buffer pH, and the use of different copper catalysts at different pH values on the described microwave-assisted Ullmann coupling reaction. We were especially interested in the question of how regioselectivity could be achieved in the presence of two nonequivalent amino groups on the aromatic system. This is an important and challenging task, especially in case of the coupling reaction of bromaminic acid (**4**) with 2,5-diaminobenzenesulfonic acid (**5**) to yield **8**, which represents a key step in the synthesis of MG 50-3-1 (**3**) [21] with typically low yield (10%) [21]. For direct comparison of the developed reaction we examined the coupling of bromaminic acid (**4**) with the isomeric 2,4-diaminobenzenesulfonic acid (**6**).

### 2.1. Optimization of the Ullmann Coupling Reaction of Bromaminic Acid with Aniline

In order to systematically optimize the microwave-catalyzed Ullmann coupling reaction [28] of bromaminic acid with anilines, we initially investigated the effects of the sodium buffer pH in the presence of four different copper catalysts having three different oxidation states (0, I and II) in a model reaction, namely the coupling reaction of bromaminic acid sodium salt (**4**) with aniline yielding Acid Blue 25 (AB-25, **7**) as outlined in Table 1. It should be noted that the pH values were measured at the start of the reaction at 23 °C (see Table 1, Table 2 and Table 4) as the reaction mixtures turned acidic during the course of the reactions due to the formation of hydrogen bromide.

**Table 1:** Impact of different pH values on the synthesis of AB-25 in the presence of a copper catalyst. ***^a^***

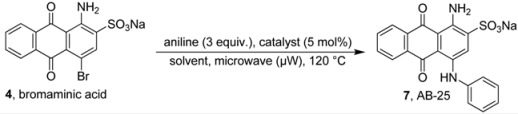											
Entry	Solvent mixture (mL) *^b^*		pH value	Cu		CuCl		CuCl_2_		CuSO_4_	
	NaH_2_PO_4 _(0.12 M)	Na_2_HPO_4 _(0.20 M)		Time (min)	Conversion (%) *^c^*	Time (min)	Conversion (%) *^c^*	Time (min)	Conversion (%) *^c^*	Time (min)	Conversion (%) *^c^*
1	Water (5)		7.0	20	ca. 50	150	0	150	0	150	0
2	5	0	4.8	25	ca. 50	150	0	150	0	150	0
3	4	1	7.0	5	100	5	100	125	ca. 50	125	ca. 50
4	3	2	7.4	5	100	5	100	65	100	35	100
5	2	3	7.8	5	100	5	100	65	100	35	100
6	1	4	8.2	5	100	5	100	35	100	35	100
7	0	5	9.4	5	100	5	100	35	100	25	100

***^a ^****Reaction conditions*: a mixture of bromaminic acid (40.5 mg, 0.1 mmol), aniline (27.5 µL, 3 eq), catalyst (5 mol %) and 5 mL of different solvent systems was irradiated under microwave conditions for 5–150 min. ***^b ^***See Table 2 and/or Table 4 for final pH values. ***^c ^***Conversion was estimated by RP-TLC using a mixture of acetone/water (1:4) as eluent; this was possible because all components (starting material and product) have different colors, bromaminic acid is red, while AB-25 is blue and the by-product is dark-red to violet.

Elemental copper (Cu) and copper(I) chloride (CuCl) gave almost the same results: they differed only in two cases, when water (pH 7, entry 1) was used as a solvent, or in acidic buffer (NaH_2_PO_4_, pH 4.8, entry 2), Cu being superior in both cases. The reaction occurred in the presence of Cu within 20–25 min with ca. 50% conversion. In the case of CuCl no conversion at all was observed in water or acidic media (pH 7 and 4.8, entry 1 and 2, respectively, Table 1), even when the mixture was harshly irradiated in the microwave oven for 150 min at 120 °C.

However, in the presence of different mixtures of phosphate buffer (entry 3-7, Table 1, neutral to basic pH values) the reaction went to completion within only 5 min (100% conversion). Next we examined the effect of the oxidation state II, represented by two different catalysts, copper(II) chloride (CuCl_2_) and copper(II) sulfate (CuSO_4_). Although both compounds are water-soluble salts and are therefore homogeneously distributed in the solution, they catalyzed the Ullmann coupling reaction less efficiently than the water-insoluble catalysts (Cu and CuCl), indicating that the Ullmann reaction can be catalyzed heterogeneously (Table 1). When water or acidic buffer were applied in the presence of a Cu^(II)^ catalyst (entry 1 and 2, Table 1) product was not detectable on an RP-TLC plate, even when harsh conditions using the microwave oven at 120 °C for 2.5 h (150 min) were applied. At more alkaline pH values (Table 1, entry 3), ca. 50% conversion was achieved (entry 3, Table 1), but the reaction took more than 2 h (125 min) in the microwave oven. Further increases in the basicity of the sodium phosphate buffer (entries 4–7, Table 1) eventually resulted in 100% conversion also for the Cu(II) catalysts, but required longer reaction times in comparison to the reactions catalyzed by Cu or CuCl.

Mechanistic studies of Ullmann reactions have previously identified Cu(I) as the single active catalytic species under various conditions [30]. However, the formation of an organometallic intermediate from elemental copper is also conceivable [31]. In a recent study we found elemental copper (Cu) to be superior to CuCl and CuSO_4_ in three different protocols in the presence or absence of microwave irradiation [28]. Our current–extended–experimental results using a simple model reaction indicate and confirm [28] that Cu^(^^0)^ appears to be the catalytic species in the performed Ullman coupling reactions.

### 2.2. Regioselective Ullmann Coupling Reaction of Bromaminic Acid with 2,5-Diaminobenzene Sulfonate

It had been previously reported that 1-amino-4-(4-amino-2-sulfophenylamino)-9,10-dioxo-9,10-dihydro–anthracene 2-sulfonate (**8**) could be obtained in 10% yield using CuCl as a catalyst in the presence of Na_2_SO_3_ in water at 40–60 °C in a reaction time of 8 h [21]. We closely followed the described procedure and tried it more than three times on different days, but all of our attempts failed, and we never observed any traces of **8**; the *meta*–isomer **9** was also not formed. The reaction did not proceed and we isolated only the starting material bromaminic acid (**4**). Then we gradually increased the temperature up to 90 °C and prolonged reaction times (up to 24 h), but without any success. Besides the starting compound **4** we now also isolated the hydrolysis product sodium 1-amino-4-hydroxyanthraquinone-2-sulfonate [28].

Therefore we performed a systematic study of the coupling of 2,5-diaminobenzene sulfonic acid (**5**) with bromaminic acid (**4**) in the presence of different catalysts and in different solvent systems with the goal to obtain product **8** (Table 2). This reaction may lead to two different isomers containing an *ortho*- (**8**) or a *meta*-sulfonate group (**9**) on ring D (Figure 1 and Table 2). At first, we examined catalysis by elemental copper, which had been identified as the most powerful catalyst in the coupling of aniline with bromaminic acid (see Table 1), in the presence of different mixtures of the phosphate buffer. As outlined in Table 2 the *meta*–sulfonate **9** (*meta* with respect to the aminoanthraquinone) was the predominant product in most cases, especially when the yield was high (>50%; entries 3–6, Table 2). The best ratio to obtain the desired *ortho*-isomer **8** (*ortho:meta* 1:1) was observed at pH 4.8 and 7.0 (entry 1 and 2, Table 2), however conversion and yield were low. Next we examined the effects of CuCl and CuSO_4_ in a sodium phosphate buffer mixture (pH 7.0, entry 8 and 9, Table 2). Within 5 min of microwave irradiation at 120 °C 100% conversion and a very good yield (>75%) was observed, but the (*ortho:meta*) ratio was dramatically reduced to 1:6.

**Table 2:** Effects of different pH values on the copper-catalyzed coupling reaction of bromaminic acid (**4**) with 2,5-diaminobenzene sulfonic acid (**5**). ***^a^***

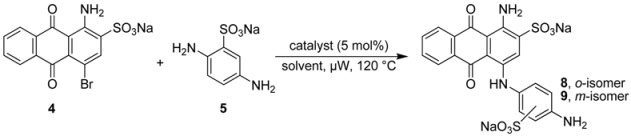								
Entry	Solvent mixture (mL)		pH value	Time (min)	Catalyst	Conversion (%) *^b^*	*ortho:meta* ratio *^b^*	Yield (%) *^c^*
	NaH_2_PO_4_ (0.12 M)	Na_2_HPO_4_ (0.20 M)						
1	5	0	4.8	10	Cu	ca. 50	1:1	ca. 25
2	4	1	7.0	5	Cu	ca. 50	1:1	ca. 25
3	3	2	7.4	5	Cu	100	1:3	> 50
4	2	3	7.8	5	Cu	100	1:3	> 50
5	1	4	8.2	5	Cu	100	1:4	> 75
6	0	5	9.4	5	Cu	100	1:4	> 75
8	4	1	7.0	5	CuCl	100	1:6	> 75
9	4	1	7.0	5	CuSO_4_	100	1:6	> 75

***^a ^****Reaction conditions*: A mixture of bromaminic acid (40.5 mg, 0.1 mmol), 2,5-diaminobenzene sulfonic acid (56.4 mg, 3 eq.), catalyst (5 mol %) and 5 mL of different buffer mixtures was irradiated in the microwave for 5–10 min at 120 °C. ***^b^*** Conversion and the *ortho:meta* sulfonate ratio was estimated by RP-TLC using a mixture of acetone/water (1:4) as eluent; this is possible because all components (starting material and product) have different colors: the starting material is red, while the product is blue and the by-product is dark-red or violet. ***^c^*** Yield was estimated based on RP-TLC results.

As a subsequent step we investigated the impact of triethylamine (Et_3_N) in water on the *ortho:meta* ratio of products **8** and **9** using three different catalysts (Table 3). The use of NEt_3_ in aqueous media is known to convert CuSO_4_ to the complex [Cu(NEt_3_)_4_]^2+^, which could then be reduced to Cu(I) [32,33].In the absence of Et_3_N the reaction was incomplete (30–60% conversion), but it was directed towards more *ortho*-isomer in the following sequence of catalysts: Cu > CuCl > CuSO_4_ (entries 1–3, Table 3). The use of three equivalents of Et_3_N improved the yield. However, the *ortho:meta* ratio was decreased; in case of Cu a ratio of 1:1 could be observed, but conversion and yield were relatively low compared to the use of CuCl and CuSO_4_ (entries 4–6, Table 3). In conclusion of this part, CuSO_4_ was found to be the best catalyst in the Ullmann coupling reaction of bromaminic acid with aniline derivative **5** in water in the presence of Et_3_N, since it showed quantitative conversion (100%) and an excellent yield (>75%) with an acceptable *ortho:meta* ratio of 1:3 (entry 6, Table 3).

**Table 3:** Effects of different copper catalysts in the presence or absence of triethylamine (Et_3_N) in aqueous medium on the synthesis of compounds **8** and **9**. ***^a^***

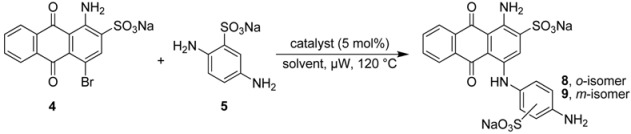						
Entry	Solvent	Time (min)	Catalyst	Conversion (%)*^b^*	*ortho:meta* ratio	Yield (%)*^c^*
1	water	15	Cu	ca. 60	3:1	ca. 50
2	water	15	CuCl	ca. 40	2:1	ca. 25
3	water	20	CuSO_4_	ca. 30	1.5:1	ca. 20
4	water/Et_3_N	5	Cu	ca. 70	1:1	ca. 40
5	water/Et_3_N	5	CuCl	ca. 90	1:3	> 50
6	water/Et_3_N	5	CuSO_4_	100	1:3	> 75

*^a^ Reaction conditions*: A mixture of bromaminic acid (40.5 mg, 0.1 mmol), 2,5-diaminobenzene sulfonic acid (56.4 mg, 3 eq.), catalyst (5 mol %) and 5 mL of water with or without Et_3_N (3 eq.) was irradiated by microwave for 5–20 min at 120 °C. *^b^* Conversion and the *ortho:meta* sulfonate ratio was estimated by RP-TLC using a mixture of acetone/water (1:4) as eluent; this is possible because all components (starting material and product) have different colors, the starting material is red, while the product is blue and the by-product is dark-red or violet. *^c^*Yield was estimated based on the RP-TLC results.

### 2.3. Regioselective Ullmann Coupling Reaction of Bromaminic Acid with 2,4-Diaminobenzene Sulfonate

For a direct comparison and to further investigate the scope of the developed Ullmann coupling reaction and its regioselectivity we reacted bromaminic acid (**4**) with 2,4-diaminobenzenesulfonic acid (**6**) using different copper catalysts. At first we examined the effect of different pH values of the sodium phosphate buffer in the presence of elemental copper (Cu) heating the reagents in a microwave oven at 120 °C for 5–10 min (see Table 4). We noticed that the reaction in the presence of Cu as a catalyst produced both possible isomers, *ortho*- and *para*-substituted.

Especially under basic conditions (pH 8.2 and 9.4, entry 5 and 6 respectively, Table 4) 100% conversion was observed and the coupling was in favour of the *para*-isomer (with respect to the aminoanthraquinone) with an *ortho:para* ratio of 1:3. The isolation of both products (**10** and **11**) was easy due to the big difference in their R*_f_* values. The purification and isolation of the two isomers proceeded by flash column chromatography using reversed phase silica gel. In neutral or acidic media the conversion was only about 25% or less (entries 1–3 Table 4) indicating the importance of basic conditions for the reaction.

**Table 4:** Effects of different pH values on the coupling reaction of bromaminic acid (**4**) with 2,4-diaminobenzene sulfonic acid (**6**). ***^a^***

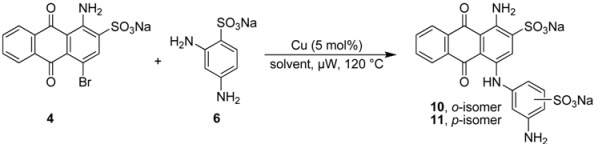							
Entry	Solvent mixture (mL)		pH value	Time (min)	Conversion (%) *^b^*	*ortho:para* ratio *^b^*	Yield (%) *^c^*
	NaH_2_PO_4_ (0.12 M)	Na_2_HPO_4_ (0.20 M)					
1	5	0	4.8	10	ca. 25	1.5:1	ca. 25
2	4	1	7.0	5	ca. 25	1:1	ca. 25
3	3	2	7.4	5	ca. 25	1:1.5	ca. 25
4	2	3	7.8	5	ca. 50	1:2	>50
5	1	4	8.2	5	100	1:3	>50
6	0	5	9.4	5	100	1:3	>50

***^a^****Reaction conditions*: A mixture of bromaminic acid (40.5 mg, 0.1 mmol), 2,5-diaminobenzene sulfonic acid (56.4 mg, 3 eq), Cu (5 mol %) and 5 mL of different buffer mixtures was irradiated by microwave for 5–10 min at 120 °C. ***^b^*** Conversion and the *ortho:para* sulfonate ratio was estimated by RP-TLC using a mixture of acetone/water (1:4) as eluent, this is possible because all components (starting material and product) have different colors: the starting material is red, while the product is blue and the by-product is dark-red or violet. ***^c^*** Yield was estimated based on the RP-TLC results.

Finally we investigated water as a solvent in the presence or absence of triethylamine using different copper catalysts: elemental copper (Cu), copper(I) chloride (CuCl), and copper(II) sulfate (CuSO_4_). The mixture was irradiated by microwave at 120 °C for 5–20 min (Table 5). Interestingly only *ortho*-isomer was detected in the water system without triethylamine with an *ortho:para* ratio of >99:1. However, the reaction did not go to completion and only 50% conversion could be observed. The addition of triethylamine dramatically increased the conversion to 100% in case of CuCl or CuSO_4 _as catalysts, whereas in the case of elemental copper (Cu) it was still only about 80%. The *ortho:para* ratio was dramatically reduced to 5:1 by applying CuCl (entry 5, Table 5). Nevertheless, the CuCl procedure was favorable due to a reaction conversion of 100%, an estimated isolated yield of >75%, and the easiness of isolating and purifying both isomers by reversed phase column chromatography.

It can thus be concluded that the best choice of catalyst in the microwave-catalyzed Ullmann coupling reaction depends on several factors, including (i) the pH value of the reaction mixture, since the protonation state of the aniline and the redox potential of the copper species are pH-dependent; (ii) the chemical nature of the reaction partners; (iii) the presence of complexing agents; and (iv) the desired regioselectivity of the reaction. Therefore the reaction conditions have to be optimized in each case, and depending on the reaction sequence a different copper species may be preferred.

**Table 5:** Effects of different copper catalysts in the presence or absence of triethylamine (Et_3_N) in water on the synthesis of compounds **8** and **9**. ***^a^***

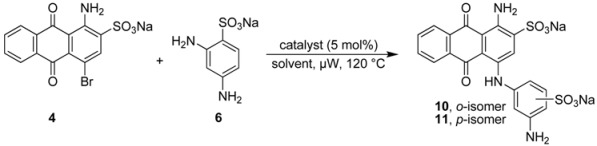						
Entry	Solvent	Time (min)	Catalyst	Conversion (%) *^b^*	*ortho:para* ratio	Yield (%) *^c^*
1	water	15	Cu	ca. 50	>99:1	ca. 20
2	water	15	CuCl	ca. 50	>99:1	ca. 20
3	water	20	CuSO_4_	ca. 50	>99:1	ca. 20
4	water/Et_3_N	5	Cu	ca. 80	1.5:1	ca. 50
5	water/Et_3_N	5	CuCl	100	5:1	>75
6	water/Et_3_N	5	CuSO_4_	100	2:1	>50

*^a^* Reaction conditions: A mixture of bromaminic acid (40.5 mg, 0.1 mmol), 2,5-diaminobenzene­sulfonic acid (56.4 mg, 3 eq), catalyst (5 mol %) and 5 mL of water with or without Et_3_N (3 eq) was irradiated by microwave for 5–20 min at 120 °C. *^b^* Conversion and the *ortho:meta* sulfonate ratio was estimated by RP-TLC using a mixture of acetone/water (1:4) as eluent. This is possible because all components (starting material and product) have different colors, the starting material is red, while the product is blue and the by-product is dark-red or violet. *^c^* Yield was estimated based on the RP-TLC results.

### 2.4. Synthesis of MG 50-3-1 (***3***)

**Scheme 1:**
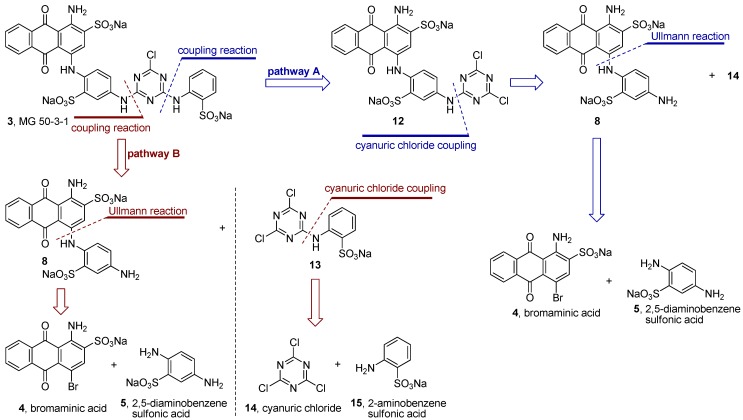
Two proposed retrosynthetic analysis pathways for MG 50-3-1 (**3**): Linear synthesis (pathway A, blue color) and convergent synthesis (pathway B, red color).

After having improved the preparation of compound **8**–the key step in the synthesis of MG 50-3-1 (**3**)–we analyzed the possible retrosynthetic pathways of **3**. Two different strategies can be envisaged: (i) a linear synthesis (pathway A, blue color, Scheme 1), which has been previously described in the literature for the synthesis of **3** (total yield of 5.5%) [21], and (ii) a new, convergent synthesis (pathway B, red color, Scheme 1).

Using the linear synthesis described in the literature (pathway A) [21] the final coupling reaction failed in our hands. The precursor of product **3** (the dichlorotriazinyl derivative **12**, Scheme 1) was found to be unstable under the conditions used for the coupling reaction (aqueous basic media), and **12** was either partially or completely hydrolyzed to the corresponding hydroxychlorotriazinyl- or dihydroxytriazinyl-anthraquinone derivative. This means that the described synthesis of MG 50-3-1 (**3**) according to pathway A failed in our hands at two critical steps, (i) the regioselective Ullmann coupling reaction described above; and (ii) the final coupling reaction step (pathway A, Scheme 1).

**Scheme 2:**
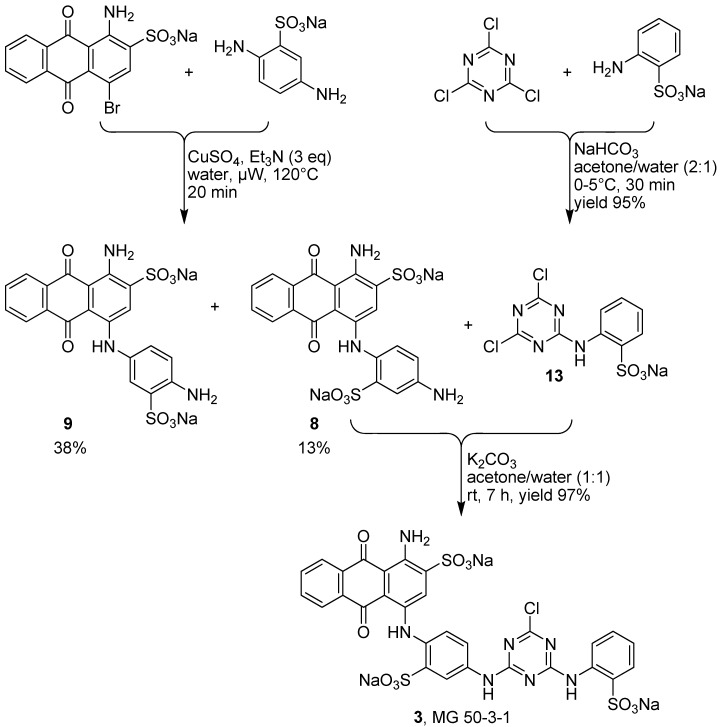
Total synthesis of MG 50-3-1 (**3**) based on a newly developed convergent synthesis (pathway B).

Therefore we applied a new, convergent approach designed by retrosynthetic analysis as outlined in Scheme 1, pathway B, and finally succeeded in obtaining compound **3**. As mentioned above we systematically improved the key step in the synthesis of compound **3**, the Ullman coupling reaction of bromaminic acid (**4**) with 2,5-diaminobenzensulfonic acid (**5**) by applying CuSO_4_ (5 mol %) as a catalyst in water in the presence of 3 equivalents of triethylamine (entry 6, Table 3). The mixture was irradiated by microwave at 120 °C for 20 min. Product **8** (the Western part of the target molecule **3**) was obtained in 13% isolated yield while its isomer **9** was obtained in 38% isolated yield (Scheme 2). The two isomers (*ortho*-isomer **8** and *meta*-isomer **9**) where readily separated by flash column chromatography on reversed phase silica gel (RP-18) using a gradient of acetone/water as described in the experimental part. The Eastern part of the target structure **3** was synthesized by the coupling of cyanuric acid chloride (**14**) with 2-aminobenzenesulfonic acid (**15**) in the presence of sodium bicarbonate (NaHCO_3_) in acetone/water (2:1) at 0–5 °C in an ice-bath for 30 min. Compound **13** was isolated in 95% yield. The last challenge was the final coupling reaction of the Western part **8** and the Eastern part **13**, which required basic conditions (K_2_CO_3_) that might lead to hydrolysis. Since we had developed a simple reaction yielding gram amounts of compound **13** with excellent yield (95%) the use of **13** in excess did not constitute any problem.

Finally **13** was successfully coupled with **8** in basic media (K_2_CO_3_) using acetone/water (1:1) as a solvent at room temperature in a reaction time of 7 h. In order to quantitatively convert the precious reagent **8**, the use of 3 equivalents of compound **13** was essential. The target compound MG 50-3-1 (**3**) was obtained in excellent isolated yield (97%). The total isolated yield over three steps was 12%.

## 3. Experimental

### 3.1. General

All solvents and reagents were used as purchased. Thin-layer chromatography was performed using TLC aluminum sheets with silica gel 60 F_254_, or TLC aluminum sheets RP silica gel 18 F_254_ (Merck, Darmstadt, Germany). Coloured compounds were visible at daylight; other compounds were visualized under UV light. Flash column chromatography was performed on a Büchi system using silica gel RP-18 (Merck, Darmstadt, Germany). ^1^H- and ^13^C-NMR data were collected on a Bruker Avance 500 MHz NMR spectrometer at 500 MHz (^1^H), or 126 MHz (^13^C), respectively. DMSO-d_6_ was used as a solvent. Chemical shifts are reported in parts per million (ppm) relative to the deuterated solvent, *i.e.*, DMSO, δ ^1^H: 2.49 ppm, ^13^C: 39.7 ppm, coupling constants *J* are given in Hertz and spin multiplicities are given as s (singlet), d (doublet), t (triplet), q (quartet), m (multiplet), br (broad). The purities of isolated products were determined by LC-ESI–MS (Applied Biosystems API 2000 LCMS/MS, HPLC Agilent 1100) using the following procedure: the compounds were dissolved at a concentration of 0.5 mg/mL in H_2_O-MeOH = 1:1, containing 2 mM NH_4_CH_3_COO. Then, 10 μL of the sample was injected into an HPLC column (Phenomenix Luna 3μm C18, 50 × 2.00 mm). Elution was performed with a gradient of water-methanol (containing 2 mM NH_4_CH_3_COO) from 90:10 to 0:100 for 30 min at a flow rate of 250 μL/min, starting the gradient after 10 min. UV absorption was detected from 200 to 950 nm using a diode array detector. Purity of the compounds was determined over the whole range (200 to 950 nm) and proved to be ≥95%. For microwave reactions a CEM Focused^TM^ Microwave Synthesis type Discover apparatus was used. A freeze dryer (CHRIST ALPHA 1-4 LSC) was used for lyophilization.

### 3.2. General Procedure A: Coupling Reaction of Bromaminic Acid with Aniline

To a 5 mL microwave reaction vial equipped with a magnetic stirring bar were added bromaminic acid sodium salt (**4**, 40.5 mg, 0.1 mmol), aniline (27.5 µL, 3 eq) and 5 mol % catalyst (Cu, CuCl, CuCl_2_, or CuSO_4_) followed by 5 mL of different solvent systems consisting of different concentrations of sodium phosphate buffer {Na_2_HPO_4_ (pH 9.4) [0.20 M] and NaH_2_PO_4_ (pH 4.8) [0.12 M]} or water (pH 7). The mixture was capped and irradiated in the microwave oven (100 W) for 5–150 min at 120 °C. Then the reaction mixture was cooled to rt, and the conversion was determined by RP-TLC using acetone/water (1:4) as the mobile phase.

### 3.3. General Procedure B: Coupling Reaction of Bromaminic Acid with 2,5-Diaminobenzensulfonic Acid (***5***) or with 2,4-Diaminobenzensulfonic Acid (***6***)

To a 5 mL microwave reaction vial equipped with a magnetic stirring bar were added bromaminic acid sodium salt (**4**, 40.5 mg, 0.1 mmol), 2,5-diaminobenzene sulfonic acid (**5**) or 2,4-diaminobenzene sulfonic acid (**6**) (56.4 mg, 3 eq), and 5 mol % catalyst (Cu, CuCl, or CuSO_4_) followed by 5 mL of different solvent systems consisting of different concentrations of sodium phosphate buffer {Na_2_HPO_4_ (pH 9.4) [0.20 M] and NaH_2_PO_4_ (pH 4.8) [0.12 M]}, water (pH 7) or water in the presence of three equivalents of triethylamine. The mixture was capped and irradiated in the microwave oven (100 W) for 5-20 min at 120 °C. Then the reaction mixture was cooled to rt, and the conversion was determined by RP-TLC using acetone/water (1:4) as eluent.

### 3.4. Synthesis of Disodium 1-Amino-4-(4-amino-2-sulfophenylamino)-9,10-dioxo-9,10-dihydro­anthracene 2-Sulfonate (***8***)

To an 80 mL microwave reaction vial equipped with a magnetic stirring bar were added bromaminic acid (**4**, 1.212 g, 3 mmol), 2,5-diaminobenzenesulfonic acid (**5**, 1.694 g, 3 eq.), CuSO_4_ (37.5 mg), Et_3_N (1.25 mL, 3 eq.) and water (45 mL) and heated in the microwave at 120 °C for 20 min, yielding a mixture of two isomers (*ortho*- and *meta*-sulfonate). The reaction mixture was cooled to rt, and the product was purified using the following procedure. The mixture was transferred to a 1000 mL separatory funnel and ca. 300 mL of water was added. The aqueous solution was extracted with dichloromethane (250 mL). The extraction procedure was repeated until the dichloromethane layer became colorless (3 times). Then the aqueous layer was reduced by rotary evaporation to ca. 20 mL, which was subsequently submitted to flash column chromatography (FCC) using RP-18 silica gel and water as an eluent. Then acetone/water (1:4) was applied at a relatively slow flow rate (10 mL·min^−1^). The first fractions containing blue product were collected. The pooled product-containing fractions were evaporated under vacuum to remove the acetone, and the remaining water was subsequently removed by lyophilization to yield the two isomers: compound **8** (210 mg, 13%) as a blue powder. The second fractions contained compound **9** (600 mg, 38%) were obtained. The total yield of both isomers (*ortho*- and *meta*-sulfonate) was 51%. Analytical data for compound **8**: mp > 300 °C, ^1^H-NMR: δ 5.14 (bs, 2H, 4′-NH_2_), 6.54 (dd, 1H, 5′-H), 6.83 (d, 1H, 6′-H), 7.13 (d, 1H, 3′-H), 7.78 (m, 2H, 6-H, 7-H), 7.88 (s, 1H, 3-H), 8.26 (m, 2H, 5-H, 8-H), 10.25 (br, 2H, 1-NH_2_), 12.00 (s, 1H, 4-NH). ^13^C-NMR: δ 109.2, 110.6, 113.4, 114.5, 124.6, 124.8, 125.8, 125.9, 126.0, 132.3, 132.5, 134.2, 134.3, 141.8, 142.1, 142.2, 144.2, 145.4, 180.0, 181.6. LC-MS (m/z): 507 [M-2Na+NH_4_^+^]^+^, 490 [M-2Na]^+^, 488 [M-2Na]^−^, 244 [M-2Na]^2−^. Purity by HPLC-UV (200 to 950 nm)-ESI-MS: 95%.

### 3.5. Synthesis of Sodium 2-(4,6-dichloro-1,3,5-triazin-2-ylamino)benzenesulfonate (***13***)

A cold solution (0–5 °C) of 2-aminobenzene sulfonic acid (**15**, 0.935 g, 5.4 mmol) in 30 mL of a mixture of acetone/water (1:1) was dropwise added to a solution of cyanuric chloride (**14**, 1.0 g, 5.4 mmol) in acetone (15 mL) with stirring at 0–5 °C. Aqueous sodium hydrogen carbonate (NaHCO_3_, 0.3 M, 12 mL) was then added to the mixture while maintaining a temperature of 0–5 °C. The solution was stirred for 30 min (TLC monitoring methanol-dichloromethane 2:3). The acetone was evaporated under vacuum, then concd. aq. HCl solution was dropwise added with cooling in an ice bath until a white precipitate formed. The white solid was collected by suction filtration and washed with water. The solid was then dried in an oven at 70 °C, while the filtrate was extracted with ethyl acetate (3 x 150 mL), and the combined organic phase was washed, once with water and dried over magnesium sulfate (MgSO_4_) and evaporated under vacuum to obtain a white precipitate. Both white solids were combined and 1.67 g of **13** were obtained (yield 90%). Analytical data: mp > 300 °C, ^1^H-NMR: δ 7.17 (ddd, 1H, 5-H), 7.44 (ddd, 1H, 4-H), 7.73 (dd, 1H, 3-H), 8.15 (dd, 1H, 6-H), 11.03 (s, 1H, 2-NH). ^13^C-NMR: δ 121.2, 124.3, 127.3, 130.0, 137.2, 154.5, 163.4. LC-MS (*m/z*): 660 [2M-Na+NH4+]^+^, 643 [2M-Na]^+^, 338 [M-Na+NH_4_^+^]^+^, 321 [M-Na^+^]^+^, 319 [M-Na]^−^, 283 [M-Na-Cl]^−^. Purity by HPLC-UV (200 to 950 nm)-ESI-MS: 98%.

### 3.6. Synthesis of Trisodium 1-Amino-4-{4-[4-chloro-6-(2-sulfophenylamino)-1,3,5-triazin-2-ylamino]-2-sulfophenylamino}-9,10-dioxo-9,10-dihydroanthracene 2-Sulfonate (***3***, *MG 50-3-1*)

A solution of sodium 2-(4,6-dichloro-1,3,5-triazin-2-ylamino)benzenesulfonate (**13**, 34,5 mg, 0.1 mmol) in 20 mL of water/acetone (1:1) was added at room temperature (rt) to a stirred solution of disodium 1-amino-4-(4-amino-2-sulfophenylamino)-9,10-dioxo-9,10-dihydroanthracene 2-sulfonate (**8**, 53.5 mg, 0.1 mmol) and K_2_CO_3_ (14 mg, 1 eq) in 20 mL of water-acetone (1:1). The resulting mixture was stirred at rt for 1 h. Control of the reaction by RP-TLC using water-acetone (1:4) as eluent showed ca. 30% conversion. Then another equivalent of **13** (34.5 mg) and K_2_CO_3_ (14 mg) was added and the resulting mixture was stirred for 2 more h. The RP-TLC analysis then showed more product (ca. 90% conversion). Finally one further equivalent of **13** (34.5 mg) and K_2_CO_3_ (14 mg) was added and the resulting mixture was stirred for additional 4 h, until the RP-TLC analysis shows >99% conversion. The reaction stopped and the acetone was evaporated under vacuum. The aqueous mixture was then subjected to FCC using RP-18 silica gel and water-acetone (1:4) as an eluent. The polarity of the mobile phase was then gradually decreased by the addition of acetone in the following steps: 5, 20, 40, and 60%. Fractions containing blue product were collected. The pooled product-containing fractions were evaporated under vacuum to remove the acetone, and the remaining water was subsequently removed by lyophilization to yield MG 50-3-1 (**3**, 81.5 mg, 97%) as blue powder in a total yield of 12%. ^1^H-NMR (DMSO-d6) δ 7.13 (s, 1H, 4′′′-H), 7.17 (m, 1H, 5′-H), 7.23 (m, 1H, 6′′′-H), 7.42 (m, 1H, 5′′′-H), 7.65 (br, 1H, 3′′′-H), 7.73 (d, 1H, 6′-H), 7.83 (m, 2H, 6-H, 7-H), 7.91 (br, 1H, 3′-H), 8.05 (s, 1H, 3-H), 8.26 (m, 2H, 5-H, 8-H), 10.43 (s, 1H, 3′′-NH), 10.50 (s, 1H, 4′-NH), 11.81 (s, 1H, 4-NH). ^13^C-NMR (DMSO-d6) δ 109.9, 113.9, 122.5, 123.9, 124.5, 124.9, 125.0, 125.4, 126.10, 126.18, 126.5, 129.6, 130.8, 131.5, 132.9, 133.2, 133.9, 134.2, 135.5, 138.1, 138.7, 139.6, 141.7, 144.7, 147.8, 155.3, 155.6, 181.9, 182.4. LC-MS (m/z): 791 [M-3Na+NH_4_^+^]^+^, 774 [M-3Na]^+^, 772 [M-3Na]^−^, 385 [M-3Na]^2−^. Purity by HPLC-UV (200 to 950 nm)-ESI-MS: 96%.

## 4. Conclusions

In conclusion, we have developed a convenient, fast, mild, and efficient convergent procedure for the synthesis of MG 50-3-1 (**3**), a potent and selective antagonist at P2Y_1_-like receptors exhibiting high potency in the low nano-molar range (IC_50_ 4.6 nM). P2Y_1_ receptors are considered as promising targets for novel anti-thrombotic drugs and for the treatment of vascular inflammation. The total synthesis of product **3** faced serious problems: a regioselective Ullmann coupling reaction was involved and successfully performed by carefully examining a variety of reaction conditions using four different copper catalysts in three different oxidation states (Cu, CuCl, CuCl_2_, and CuSO_4_) and by investigating the microwave-assisted reaction at different pH values. Results show the combination of CuSO_4_ in water as solvent and in the presence of triethylamine to provide the optimal conditions toward the synthesis of MG 50-3-1 precursor **8**. The second challenge was the hydrolysis of the chlorotriazinyl moiety; we were able to eliminate this problem by synthesizing the new synthon **13**, which could easily be obtained on a gram scale. It should be mentioned that compound **13** was also partially hydrolyzed during the reaction, but the use of three equivalents of the easily accessible **13** led to complete conversion of all starting material **8** yielding the target molecule (MG 50-3-1, **3**) in 97% isolated yield. 

## References

[1] Burnstock G. (2007). Physiology and pathophysiology of purinergic neurotransmission. Physiol. Rev..

[2] Burnstock G. (2006). Pathophysiology and therapeutic potential of purinergic signaling. Pharmacol. Rev..

[3] Müller C.E. (2002). P2-pyrimidinergic receptors and their ligands. Curr. Pharm. Des..

[4] Brunschweiger A., Müller C.E. (2006). P2 receptors activated by uracil nucleotides—An update. Curr. Med. Chem..

[5] Hancock A.A. (2006). The challenge of drug discovery of a GPCR target: Analysis of preclinical pharmacology of histamine H3 antagonists/inverse agonists. Biochem. Pharmacol..

[6] Burnstock G. (2002). Purinergic signaling and vascular cell proliferation and death. Arterioscler. Thromb. Vasc. Biol..

[7] Gachet C. (2008). P2 receptors, platelet function and pharmacological implications. Thromb. Haemost..

[8] Gachet C. (2005). The platelet P2 receptors as molecular targets for old and new antiplatelet drugs. Pharmacol. Ther..

[9] Gachet C. (2006). Regulation of platelet functions by P2 receptors. Annu. Rev. Pharmacol. Toxicol..

[10] Cattaneo M. (2006). P2Y_12_ receptor antagonists: A rapidly expanding group of antiplatelet agents. Eur. Heart J..

[11] Cattaneo M. (2004). Aspirin and clopidogrel efficacy, safety, and the issue of drug resistance *Arterioscler*. Thromb. Vasc. Biol..

[12] Nawarskas J.J., Clark S.M. (2011). Ticagrelor: A novel reversible oral antiplatelet agent. Cardiol. Rev..

[13] Léon C., Hechler B., Freund M., Eckly A., Vial C., Ohlmann P., Dierich A., LeMeur M., Cazenave J.-P., Gachet C. (1999). Defective platelet aggregation and increased resistance to thrombosis in purinergic P2Y_1_ receptor-null mice. J. Clin. Invest..

[14] Fabre J.E., Nguyen M., Latour A., Keifer J.A., Audoly L.P., Coffman T.M., Koller B.H. (1999). Decreased platelet aggregation, increased bleeding time and resistance to thromboembolism in P2Y_1_-deficient mice. Nat. Med..

[15] King B.F., Townsend-Nicholson A.J. (2008). Involvement of P2Y_1_ and P2Y_11_ purinoceptors in parasympathetic inhibition of colonic smooth muscle. Pharmacol. Exp. Ther..

[16] Bean B.P. (1992). Pharmacology and electrophysiology of ATP-activated ion channels. Trends Pharmacol. Sci..

[17] Inoue K., Nakazawa K., Ohara-Imaizumi M., Obama T., Fujimori K., Takanaka A. (1991). Antagonism by reactive blue 2 but not by brilliant blue G of extracellular ATP-evoked responses in PC12 phaeochromocytoma cells. Br. J. Pharmacol..

[18] Nakazawa K., Inoue K., Fujimori K., Takanaka A. (1991). Effects of ATP antagonists on purinoceptor-operated inward currents in rat phaeochromocytoma cells. Pflügers Arch..

[19] Brown J., Brown C.A. (2003). Evaluation of reactive blue 2 derivatives as selective antagonists for P2Y receptors. Vasc. Pharmacol..

[20] Tuluc F., Bültmann R., Glänzel M., Frahm A.W., Starke K. (1998). P2-receptor antagonists: IV. Blockade of P2-receptor subtypes and ecto-nucleotidases by compounds related to reactive blue 2. Naunyn Schmiedeberg’s Arch. Pharmacol..

[21] Glänzel M., Bültmann R., Starke K., Frahm A.W. (2005). Structure-activity relationships of novel P2-receptor antagonists structurally related to Reactive Blue 2. Eur. J. Med. Chem..

[22] Baqi Y., Hausmann R., Rosefort C., Rettinger J., Schmalzing G., Müller C.E. (2011). Discovery of potent competitive antagonists and positive modulators of the P2X2 receptor. J. Med. Chem..

[23] Weyler S., Baqi Y., Hillmann P., Kaulich M., Hunder A.M., Müller I.A., Müller C.E. (2008). Combinatorial synthesis of anilinoanthraquinone derivatives and evaluation as non nucleotide-derived P2Y_2_ receptor antagonists. Bioorg. Med. Chem. Lett..

[24] Baqi Y., Atzler K., Köse M., Glänzel M., Müller C.E. (2009). High-affinity, non nucleotide-derived competitive antagonists of platelet P2Y_12_ receptors. J. Med. Chem..

[25] Hoffmann K., Baqi Y., Morena M.S., Glänzel M., Müller C.E., von Kügelgen I. (2009). Interaction of new, very potent non-nucleotide antagonists with Arg256 of the human platelet P2Y_12_-receptor. J. Pharmacol. Exp. Ther..

[26] Baqi Y., Weyler S., Iqbal J., Zimmermann H., Müller C.E. (2009). Structure-activity relationships of anthraquinone derivatives derived from bromaminic acid as inhibitors of ectonucleoside triphosphate diphosphohydrolases (E-NTPDases). Purinerg. Signal..

[27] Baqi Y., Lee S.-Y., Iqbal J., Ripphausen P., Lehr A., Scheiff A.B., Zimmermann H., Bajorath J., Müller C.E. (2010). Development of potent and selective inhibitors of ecto-5’-nucleotidase based on an anthraquinone scaffold. J. Med. Chem..

[28] Baqi Y., Müller C.E. (2007). Rapid and efficient microwave-assisted copper(0)-catalyzed Ullmann coupling reaction: general access to anilinoanthraquinone derivatives. Org. Lett..

[29] Baqi Y., Müller C.E. (2010). Synthesis of alkyl- and aryl-amino-substituted anthraquinone derivatives by microwave-assisted copper(0)-catalyzed Ullmann coupling reactions. Nat. Protoc..

[30] Paine A.J. (1987). Mechanisms and models for copper mediated nucleophilic aromatic substitution. 2. A single catalytic species from three different oxidation states of copper in an Ullmann synthesis of triarylamines. J. Am. Chem. Soc..

[31] Lewin A.H., Cohen T. (1965). The mechanism of the Ullmann reaction. Detection of an organocopper intermediate. Tetrahedron Lett..

[32] Ritter S.C. (2007). Cu(I)-Catalyzed “Click-Chemistry“ Design of a Chemical Photomultiplier Target-Guided Synthesis of Bidentate Metal-Complex Receptors. Ph.D. thesis.

[33] Ritter S.C., König B. (2006). Signal amplification and transduction by photo-activated catalysis. Chem. Commun..

